# Is weight-based IV dosing of trastuzumab preferable to SC fixed-dose in some patients? A systematic scoping review

**DOI:** 10.1016/j.breast.2021.03.003

**Published:** 2021-03-18

**Authors:** Hans-Christian Kolberg, Christian Jackisch, Sara A. Hurvitz, Julie Winstone, Helen Barham, Vladimir Hanes, Delphine Courmier

**Affiliations:** a Department of Gynecology and Obstetrics, Breast and Gynecologic Cancer Center, Marienhospital, Josef-Albers-Str. 70, 46236, Bottrop, Germany; b Department of Obstetrics and Gynecology, Breast and Gynecologic Cancer Center, Sana Klinikum Offenbach, Starkenburgring 66, D-63069, Offenbach, Germany; c Department of Medicine, Division of Hematology/Oncology, University of California, Los Angeles, Jonsson Comprehensive Cancer Center, Los Angeles, CA, USA; d Perscribo Medical Communications Ltd, 7 York Close, Kempshott Rise, Basingstoke, RG22 4PU, UK; e Amgen Biosimilar Business Unit, Amgen Inc., One Amgen Center Drive, Thousand Oaks, CA 91320USA, USA; f Amgen Global Health Economics, Amgen Inc., One Amgen Center Drive, Thousand Oaks, CA 91320USA, USA

**Keywords:** Trastuzumab, Breast cancer, Subcutaneous, Pharmacokinetics, Fixed-dose, Body weight, ADA, antidrug antibody, ARR, administration-related reaction, EBC, early breast cancer, ISR, injection site reaction, JBI, Joanna Briggs Institute, mAb, monoclonal antibody, MBC, metastatic breast cancer, MGC, metastatic gastric cancer, popPK, population pharmacokinetics, rHuPH20, recombinant human hyaluronidase, RWE, real-world evidence, TEAE, treatment-emergent adverse event

## Abstract

Trastuzumab, a key treatment for HER2-positive breast cancer, is available in weight-based IV and fixed-dose (600 mg) SC formulations. While the Phase 3 HannaH trial indicated non-inferiority of the SC formulation, there is some concern that the target plasma concentration may not be reached in overweight/obese patients whereas low-body-weight patients may be at risk of toxicity.

This scoping review evaluated whether overweight/obese patients are at risk of below-target exposure with fixed-dose SC trastuzumab, whether low-body-weight patients are at risk of increased toxicity, especially cardiotoxicity, and whether IV and SC trastuzumab are equivalent in terms of treatment-emergent adverse events (TEAEs) (e.g. infections). Thirty-seven publications that met the eligibility criteria were included.

Body weight is not an important determinant of exposure to trastuzumab at steady state (i.e. pre-dose cycle 8); however, real-world evidence suggests that the target concentration (20 μg/mL) may not be reached with the first SC dose in overweight/obese patients. There is no evidence that low-body-weight patients are at increased risk of cardiotoxicity with SC trastuzumab, although this may be confounded by the higher rate of cardiovascular comorbidities in overweight patients. In Phase 3 trials, SC trastuzumab was associated with higher rates of ISRs, ADAs and SAEs, the latter often requiring hospitalization and occurring during adjuvant treatment when patients are not burdened by chemotherapy.

The route of administration of trastuzumab (IV vs SC) in different treatment settings should be discussed with the patient, taking into account the risks and benefits associated with each route.

## Highlights

• A systematic scoping review of fixed-dose SC trastuzumab and body weight.
• Trastuzumab levels may not reach target with the first dose in overweight patients.
• Low body weight does not seem to increase cardiotoxicity risk with SC trastuzumab.
• Adverse event rates were higher with SC than IV trastuzumab in Phase 3 trials.
• The risks and benefits of IV vs SC trastuzumab should be discussed with the patient.

## 1 Introduction

Trastuzumab has been a mainstay in the treatment of HER2-positive breast cancer for 30 years. Until recently it was administered by IV infusion at a dose based on body weight, an approach widely used in oncology to overcome interindividual variability in drug exposure [1]. However, a fixed-dosed SC formulation (Hylecta™; Roche) was introduced the EU in 2013 and has recently become available in the USA.

### 1.1 Development of the IV weight-based dosing strategy

Optimizing the therapeutic index across a target patient population requires understanding of the exposure–effect relationship and interindividual variability in the PK of the therapeutic [2]. The optimal efficacy of monoclonal antibodies (mAbs) requires saturation of the accessible target sites [3]. The minimum target serum trough concentration (C_trough_) for trastuzumab is 20 μg/mL, based on studies in xenograft models and early clinical response data in patients with metastatic breast cancer (MBC) [4]. A population model for IV trastuzumab indicated that HER2 receptors are saturated in the clinical concentration range on the basis of linear PK, with a subsequent plateau in the dose–efficacy relationship [3].

Whilst studies of IV trastuzumab demonstrated that fixed weekly doses ≥250 mg achieved the target C_trough_ of >20 μg/mL, with linear PK, a weight-based regimen was adopted after two phase 2 trials (H0551g and H0552g) suggested that intersubject variability in trastuzumab PK was related to body weight. Population PK (popPK) analysis showed that C_trough_ 20 μg/mL was achieved in approximately 90% of patients at the approved IV dose, with a mean C_trough_ of 25.0 μg/mL after the first dose [5].

### 1.2 SC versus IV administration

SC trastuzumab contains recombinant human hyaluronidase (rHuPH20) to enhance drug absorption and is administered as a fixed dose (600 mg) over 2–5 min [6]. This dose was determined from modeling and simulation using PK data from the Phase 1 dose-finding study [[7], [8], [9]]. The comparability of the IV and SC formulations in terms of efficacy and PK has been demonstrated in the non-inferiority HannaH trial [[10], [11], [12], [13]].

Whilst PK modeling for mAbs showed similar PK variability with weight-based and fixed doses [14], a PK model for trastuzumab indicated that fixed dosing resulted in greater variability and deviation than weight-based dosing [15]. In their initial review of SC trastuzumab, the FDA noted that transition to fixed dosing from weight-based dosing could lead to under- or over-dosing of patients in the extremes of the body weight spectrum, referring to the popPK model demonstrating that body weight was a significant covariate [16]. This may be a concern in some countries given the prevalence of overweight/obesity, and that obesity is an established risk factor for breast cancer in postmenopausal women [17]. For example, the age-adjusted prevalence of obesity among women aged over 40 years in the USA in 2017–18 was 43.3% [18]. In a US study of adjuvant trastuzumab in HER2-positive early breast cancer (EBC), 43% of 3017 patients were obese (BMI ≥ 30.0 kg/m^2^) at baseline [19]. The average BMI in women varies widely, for example from 21.8 kg/m^2^ in Japan, approximately 24 kg/m^2^ in Switzerland and France, to 29.0 kg/m^2^ in the USA [20].

The FDA review concluded that body-size based dosing is not required as SC trastuzumab achieved equal or higher C_trough_ at pre-dose cycle 8 with no worse efficacy across body weight groups compared with IV trastuzumab [16], as reflected in the current US Prescribing Information [21]. Nevertheless, we wanted to explore whether the “one size fits all“ strategy adopted with SC trastuzumab is appropriate, particularly given that in the real-world setting a broader range of patients is encountered than in clinical trials. We also compared the safety and immunogenicity of the two formulations.

### 1.3 Choice of method

A scoping review was considered to be more appropriate than a formal systematic review because it is less restrictive – the search strategy is developed iteratively, allowing additional search terms and sources identified during the review process to be incorporated into the final search strategy. As the quality of included sources is not assessed against rigorous criteria, evidence from a variety of sources and study designs can be included. Scoping reviews are ideal for evaluating broader questions than would be possible with a systematic review, such as the nature of the available evidence for a particular intervention [22]. Indeed, scoping reviews are an ideal way to map the literature and identify more specific questions that might be subsequently addressed through a full systematic literature review [23]. The importance of the scoping review as a valid and accepted approach is recognized by Cochrane and PRISMA [22,24].

Direct evidence (e.g., efficacy data from SC trastuzumab studies) was used wherever possible. In the absence of direct evidence, indirect evidence was used, including popPK, exposure–response analyses, and data from dose-escalation studies of IV trastuzumab.

### 1.4 Objectives

The overall objective was to evaluate the appropriateness of fixed-dose SC trastuzumab across all patients with HER2-positive breast cancer. Three questions were considered:

• Are overweight/obese patients at risk of sub-target exposure with fixed-dose SC trastuzumab?
• Are low-body-weight patients at risk of increased toxicity, especially cardiotoxicity?
• Are the two formulations equivalent in terms of TEAEs?

## 2 Method

The general principles outlined in the Joanna Briggs Institute (JBI) Reviewer’s Manual for conducting a scoping review [25] were followed. The full method is reported in the Supplementary appendix. The objectives of the scoping review were addressed using the PICOS (population, intervention, comparators, outcomes, study design/setting) criteria. The inclusion and exclusion criteria were developed separately for each review question (Supplementary Tables 1–3).

The search strategy was developed iteratively, as per the JBI-recommended three-step approach, allowing incorporation of search terms and sources identified during the review process [25]. Studies were identified by searching electronic databases, reference lists of relevant articles, regulatory agency websites, and conference proceedings (via the Conference Proceedings Citation Index). The core search strategy is provided in Supplementary Table 4, together with modifications to account for differences in syntax and thesaurus headings for searches of the Cochrane Library, SCOPUS and Web of Science (Supplementary Table 5–7).

The search results and citation screening were managed in an EndNote® library. Titles and abstracts (when available) were reviewed by two independent researchers in a standard two-pass review process. Final inclusion and exclusion was verified by the lead researcher. The final list of studies for inclusion in the review was agreed by the study team.

Data from eligible studies were extracted into a spreadsheet by the lead researcher and quality checked by an independent reviewer. Extracted study-level data included publication details, study, demographic and treatment characteristics, PK and efficacy outcomes, AEs (including cardiotoxicity), and rate of antidrug antibodies (ADAs). The findings were tabulated and a narrative synthesis developed, as per the guidelines [25].

## 3 Results and discussion

**Fig. 1:**
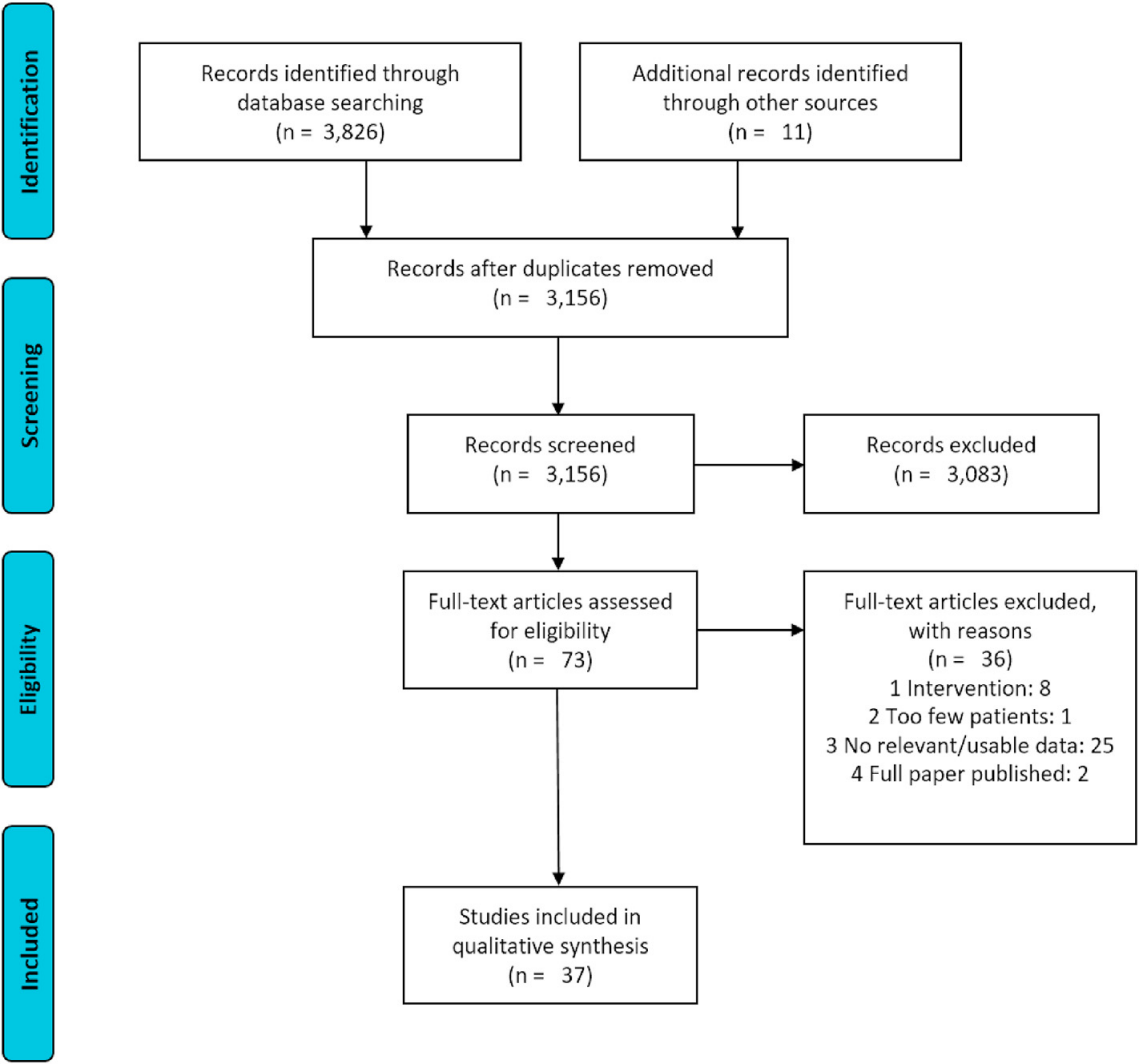
PRISMA diagram, illustrating the flow of articles though the scoping review. Adapted from [26].

The literature search identified 3826 unique citations and a further 11 were identified from the grey literature/hand search. Thirty-seven publications met the eligibility criteria after de-duplication, screening and full text review, and were included in the scoping review; the PRISMA flow chart is shown in Fig. 1.

**Question 1: Are overweight/obese patients at risk of sub-target exposure with fixed-dose SC trastuzumab?**

Relevant information was identified from 16 articles and two regulatory reports. Data directly comparing SC and IV trastuzumab by body weight or PK exposure come from the HannaH study, a Phase 3 non-inferiority study involved 596 patients with stage 1–3 HER2-positive EBC, who received neo/adjuvant trastuzumab once every 3 weeks, either SC (600 mg) or IV (8 mg/kg loading dose; 6 mg/kg maintenance dose) [10]. The co-primary endpoints were serum trough concentration of trastuzumab before surgery (C_trough_ pre-dose cycle 8; i.e. steady state) and the pathologic complete response (pCR) rate; the PK profile was a secondary endpoint. Non-inferiority of SC versus IV trastuzumab was reported for both primary endpoints at 12 months [10], 20 months [12], 2 years [11] and 6 years [13]. Exploratory analyses at 20 months did not identify any associations between pCR or event-free survival (secondary endpoint) and body weight or exposure with SC trastuzumab [12].

PopPK models are widely used to determine the influence of different covariates, including body weight, on interindividual variability in PK [2]. PopPK models for trastuzumab consistently identified body weight [7,15,[27], [28], [29], [30], [31], [32]] or BMI [33] as a significant covariate for linear clearance (point estimate > 0.5 [1]) in HER2-positive EBC, MBC and metastatic gastric cancer (MGC). These are summarized in Supplementary Appendix Table 8.

Quartino and colleagues reported a popPK model for SC and IV trastuzumab based on the HannaH data; multiple logistic regression analysis determined that body weight did not affect the primary efficacy endpoint. Estimated odds ratios were similar across both weight and exposure quartiles, prompting the authors to conclude that dose adjustment for patient factors was not required within the ranges studied [7]. However, the point estimate for body weight on CL was 1.04, and body weight explained 8% of the total variability in linear clearance, 10% in the central volume of distribution, and 28% in peripheral volume of distribution [12]. Interpatient variability in clearance is particularly relevant because this strongly influences the serum concentration at the end of the dosing interval [34]. The CHMP reported that 92% of patients weighing ≥90 kg achieved steady-state C_trough_ levels (>20 μg/mL), compared with 100% after weight-based IV dosing, and also concluded that an approximately bioequivalent SC dose based on steady state would be 8 mg/kg, which corresponds to fixed doses of about 400 mg, 600 mg and 750 mg for patients weighing <51, 51–90 and > 90 kg, respectively [35].

The authors of the HannaH report highlight the importance of exceeding the target trastuzumab concentration of 20 μg/mL after the first dose, in order to avoid the need for a loading dose [10]. However, the HannaH PK analysis was based on plasma levels at pre-dose cycle 8 (i.e., at steady state, near the end of neoadjuvant treatment) and does not report whether target concentrations of trastuzumab were achieved during the first cycle.

A Phase 1/1b study of weight-based dosing in healthy volunteers (n = 66) reported that 8 mg/kg SC trastuzumab was likely to achieve comparable serum levels at 22 days (end of cycle 1) as 6 mg/kg IV [8]. The mean BMI of patients in the 8 and 12 mg/kg groups was 28.7 and 26.8 kg/m^2^, respectively (range 17.3–48.3 kg/m^2^). Mean C_trough_ at day 22 was 37.8 μg/mL with 8 mg/kg SC (60.8 μg/mL with 12 mg/kg SC).

Wynne also reported a simulated PK study in patients >75 kg (>75th percentile of the virtual population) comparing 600 mg SC and the approved IV dose. Simulated median C_trough_ values for cycle 1 were 36 mg/L (i.e. 36 μg/dL) (5th–95th percentile 33–39 mg/L) and 38 mg/L (34–44 mg/L) respectively, and at cycle 7 were 69 mg/L (33–130 mg/L) and 53 mg/L (25–99 mg/L), respectively, indicating similar plasma levels with the two dosage regimens [9].

The median body weight used in the popPK model simulations was 68 kg (i.e., median body weight of the SC arm in HannaH) [7], which is lower than in patients typically seen in clinical practice. For example, >60% of a clinical sample of 1041 patients were overweight or obese (BMI >30 kg/m^2^) and > 20% weighed >80 kg [36]. Wynne and colleagues subsequently reported that 29% of the patients weighed >79 kg [9], in line with these real-world data. Nevertheless, a 600 mg SC dose in an 80 kg patient is equivalent to 7.5 mg/kg, which is less than the lower dose evaluated in the Phase 1 study.

**Fig. 2:**
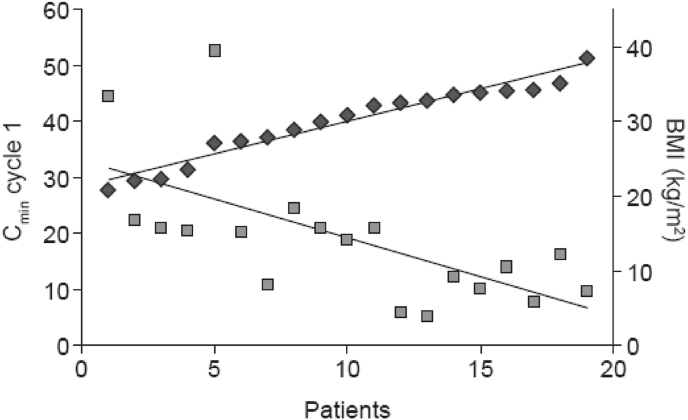
Relationship between BMI and C_min_ during the first treatment cycle with SC trastuzumab in patients with HER2-positive early breast cancer in real-world practice. Adapted from [37]

Two real-world studies in HER2-positive EBC looked at the PK from the first dose, and suggested that fewer patients achieve target trastuzumab levels during cycle 1 after SC administration than implied by HannaH findings and that this is influenced by body weight and BMI [37,38]. The HannaH study reported steady-state PK (pre-dose 8) data but not cycle 1 exposure [7,[10], [11], [12], [13]]. The target trastuzumab level in the real-world studies was C_min_ ≥20 μg/mL as established in the early studies. In the first study (n = 19), initial (pre-dose cycle 2) exposure (C_min_) with SC trastuzumab decreased with higher body weight, such that half of patients weighing 65–79 kg achieved the target concentration after the first dose of SC trastuzumab, but none of the patients weighing ≥80 kg [37]. Similarly, the target was reached in 89% of patients with BMI ≤30 kg/m^2^ but only 10% of patients with BMI >30 kg/m^2^. Median body weight in this study was 75.9 kg (Fig. 2). The mean C_min_ was lower in this study than in HannaH (19.0 [SD 12.1] vs 32.7 [18.5] μg/mL), which the authors attributed to the difference in body weight (75.9 vs 68.0 kg), indicating that weight and BMI influence trastuzumab PK [37].

**Fig. 3:**
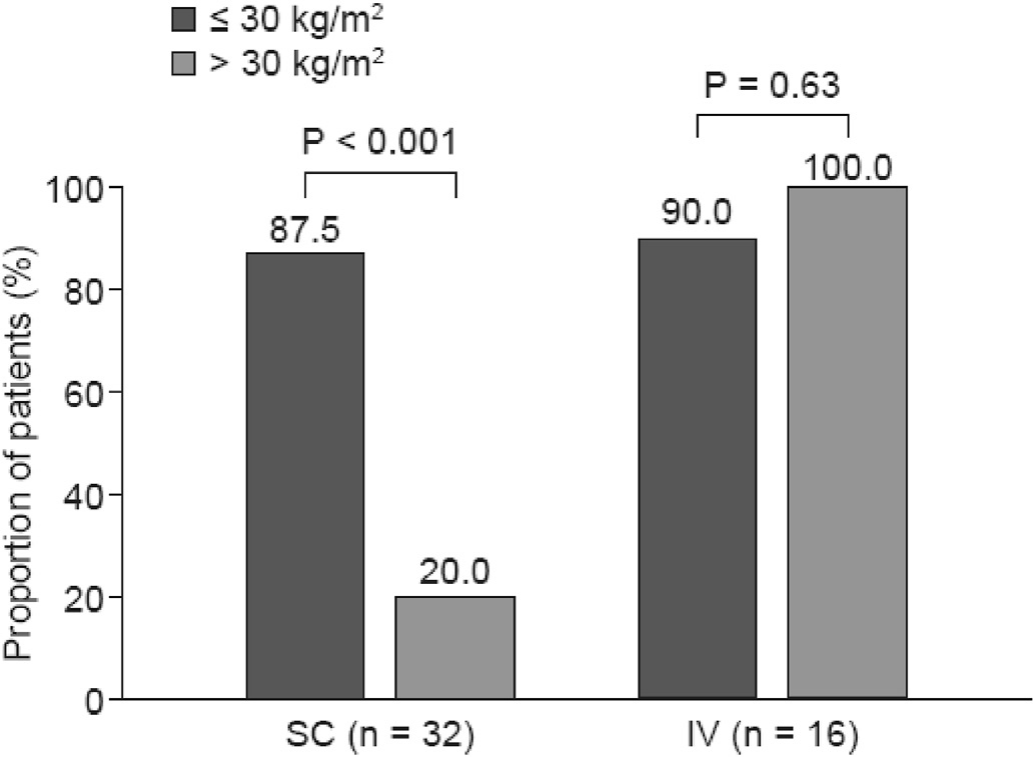
Proportion of patients achieving target exposure in cycle 1 with SC vs IV trastuzumab by BMI in real-world practice. Target exposure was Cmin before cycle 2 ≥ 20 μg/mL. Adapted from [38].

In the second study (n = 50), patients receiving weight-based dosing with IV trastuzumab had significantly higher initial concentrations of trastuzumab than those receiving fixed-dose (600 mg) SC trastuzumab. Significantly more patients in the IV group than in the SC group achieved the target concentration during the first treatment cycle (93.8% vs 67.6%; P = 0.042) [38]. As in the earlier study, body weight influenced whether the target exposure was reached with IV dosing (87.5% for patients with BMI ≤30 kg/m^2^ versus vs 20% with those >30 kg/m^2^ P < 0.001) but was not important with SC dosing (Fig. 3). Additional analyses identified BMI as the only covariate that had a significant impact on cycle 1 trastuzumab C_min_ (P < 0.05). The authors therefore concluded that the PK profiles of fixed-dose (600 mg) and weight-based IV trastuzumab are not equivalent, particularly in obese patients [38]. However, these were small studies (n = 19 and 50) conducted by the same research group, so the generalizability is not clear.

Evidence suggesting that low initial (cycle 1) plasma levels may compromise the efficacy of trastuzumab come from studies in HER2-positive MGC/gastroesophageal cancer [30,[39], [40], [41], [42]], for which IV (but not SC) trastuzumab is indicated. PopPK and exposure–response analysis of data from the Phase 3 ToGA trial (which evaluated the addition of IV trastuzumab to chemotherapy in patients with HER2-positive MGC) found that patients with progressive disease had lower exposure to trastuzumab (based on median AUC, C_max_ and C_min_) than patients who had had a partial or complete response or stable disease, although the 1.5 interquartile ranges overlapped [30]. The proportion of patients with progressive disease was highest in the lowest C_min_ quartile (<17.3 μg/mL; Table 1) and survival was markedly shorter in patients with the lowest C_min_ (≤25th percentile) at cycle 1 compared with the highest centile (overall survival [OS] 7.7 vs 13.8 months) [30]. Whilst patients in the lowest quartile also had the worse risk profile, a case–control comparison that matched patients in the lowest C_min_ quartile with patients in the control arm of ToGA (who did not receive trastuzumab) found that the risk factors were no longer significant. Median OS was similar in the two groups, suggesting that trastuzumab conferred no survival benefit when C_min_ was <11.8 μg/mL during the first treatment cycle. Whilst it is not appropriate to extrapolate efficacy findings from gastric to breast cancer, these PK results nevertheless indicate that early adequate exposure to trastuzumab may be important in terms of outcomes, which would need to be explored in breast cancer specifically.

**Table 1:** Proportions of patients in each response category steady state C_min_ quartile in the ToGA trial in MGC.

C_min_ quartile (μg/mL)	Complete response	Partial response	Stable disease	Progressive disease
<17.3	5.6	40.7	22.2	31.5
≥17.3 and < 27.6∗	5.5	49.1	36.4	9.1
≥27.6 and < 36.9∗	5.5	49.1	36.4	9.1
≥36.9	8.5	55.9	27.1	8.5

Values are % of patients in each C_min_ (minimum plasma concentration) quartile who achieved each level of response.

∗Reported per published report although one row may be incorrect as it seems unlikely that they would be identical.

Source [30].

The FDA subsequently recommended a post-marketing trial in patients with MGC and higher tumor burden, to determine whether a higher dose of trastuzumab (with acceptable safety) would result in an acceptable OS benefit [39]. The Phase IIb HELOISE trial (n = 248) confirmed that a higher maintenance dose of trastuzumab (10 mg/kg every 3 weeks following the initial 8 mg/kg loading dose) achieved higher serum concentrations than the standard maintenance dose (6 mg/kg) but with no increase in efficacy [41]. However, the ability of this study to distinguish between low trastuzumab exposure and high disease burden as the cause of lower OS in ToGA has been strongly criticized [42] and the issue remains unresolved. The manufacturer of SC trastuzumab has not sought approval for gastric cancer indications because of PK differences expected in this patient population (i.e., lower trastuzumab exposure in MGC patients) [16].

Overall, the clinical trial evidence indicates that body weight does not affect exposure to trastuzumab once steady-steady PK are reached. Importantly, however, real-world evidence suggests that target serum concentrations (20 μg/mL) may not be reached with the first 600 mg SC dose in patients who are overweight or obese – an important consideration when switching from IV to SC dosing without a loading dose [10].

**Question 2: Do patients with low body weight have the same risk for cardiotoxicity with SC and IV trastuzumab?**

Early reports of increased cardiotoxicity with trastuzumab were largely attributed to co-administration with anthracyclines, and rates are generally lower in monotherapy trials; however, concern about the potential risk of cardiotoxicity with trastuzumab and other HER2-targeted agents persists [43,44]. We therefore explored whether low-body-weight patients are at increased risk of cardiotoxicity with the 600 mg SC dose.

Relevant information was identified from 15 articles reporting 12 studies of wide-ranging designs. In the HannaH study, steady-state AUC was about 80% higher with SC than with IV trastuzumab in patients <50 kg [4,12] but exploratory analyses did not identify any clinically meaningful association between exposure and the incidence of grade ≥3 adverse events (AEs) or serious AEs (SAEs) [12] or body weight [11,13] (Table 2).

**Table 2:** Effect of body weight on Grade ≥3 AEs, SAEs, and cardiac AEs in the Phase 3 HannaH study of SC vs IV trastuzumab.

	Grade ≥3 AE, n (%)^a^		SAEs, n (%)^a^		Cardiac AEs, n/N (%)^b,^^c^	
Body weight (kg)	SC (n = 297)	IV (n = 298)	SC (n = 297)	IV (n = 298)	SC (n = 297)	IV (n = 298)
<59	37 (52%)	50 (65%)	11 (15%)	15 (19%)	6/71 (8.5%)	9/77 (11.7%)
≥59 to <68	37 (53%)	42 (50%)	15 (21%)	8 (10%)	8/70 (11.4%)	9/84 (10.7%)
≥68 to <79	42 (59%)	31 (44%)	17 (24%)	13 (19%)	14/71 (19.7%)	6/70 (8.6%)
≥79	43 (51%)	33 (49%)	21 (25%)	6 (9%)	14/85 (16.5%)	16/67 (23.9%)

AE, adverse event; AUC, area under plasma concentration–time curve; IV, intravenous; SC, subcutaneous; SAE, serious AE.

Sources: ^a^ [12]; ^b^ [11]; ^c^ [13].

Similarly, rates of grade ≥3 and cardiac AEs were not increased in lower-weight patients receiving SC trastuzumab in the SafeHer (single-arm SC trastuzumab) study [45].

In its multidisciplinary review of SC trastuzumab, the FDA commented that while the frequency of treatment-emergent cardiac AEs in the HannaH study was numerically highest in the highest weight quartile in the SC arm, the rate was similar in the highest weight quartile in the IV arm [16]. Similarly, in the SafeHer study, the incidence of treatment-emergent cardiac AEs was higher in the highest versus the lowest weight quartile [45]. The FDA suggested that this reflects the known comorbidities and risk associated with the highest weight quartile and that, overall, patients in the lowest weight quartile did not have an increased rate of treatment-emergent cardiac AEs [16]. A retrospective study (n = 260) found that BMI was not a risk factor for cardiotoxicity with SC trastuzumab [46].

Because obesity is associated with an increased risk for cardiotoxicity with both anthracyclines and trastuzumab [47], any potential increase in cardiotoxicity in low-weight patients receiving fixed-dose SC trastuzumab will be confounded by the higher rate of cardiotoxicity in obese patients. We therefore identified publications of early phase trastuzumab dose-escalation studies (Supplementary Table 9) and studies comparing high versus standard loading doses of IV trastuzumab (Supplementary Table 10), in order to investigate whether the rate of cardiotoxicity is increased with higher doses of trastuzumab. The five studies did not provide evidence of any increase in the rate of cardiotoxicity with higher doses of trastuzumab as monotherapy [8,48] or before/after chemoradiotherapy [[49], [50], [51]]. However, by the nature, these studies are short, restrict the maximum administered trastuzumab dose, and include few patients at each dose level. The maximum dose was likely guided by the target C_trough_ for therapeutic effectiveness reported in the Phase 1 studies of IV monotherapy in MBC (H0407g and H0453g) but these have not been published so it is not known whether overdosing of trastuzumab per se was associated with an increased frequency of cardiotoxicity.

Three studies showed that higher loading doses of trastuzumab were not associated with an increased frequency of cardiac AEs [41,52,53]. However, any potential increase in rate of cardiotoxicity in low-weight patients receiving fixed-dose SC trastuzumab could be confounded by the higher rate of cardiotoxicity in obese patients.

Overall, this scoping review did not identify any evidence suggesting that patients of low-body-weight have an increased risk of cardiotoxicity with 600 mg SC trastuzumab.

**Question 3: Is the risk of adverse events and immunogenicity the same for patients receiving SC and IV trastuzumab?**

Relevant information was identified in 14 articles reporting five studies, including the EMA and FDA regulatory reports for SC trastuzumab and one rapid (systematic) review evaluating the efficacy and safety of SC and IV trastuzumab.

### 3.1 Overall toxicity

Data from the pivotal HannaH study showed similar rates of any-grade AEs with SC and IV trastuzumab (97% vs 94%) [10], and grade ≥3 AEs (54% vs 52%), with a numerically higher rate of treatment discontinuation because of AEs with SC trastuzumab (5.7%, vs 2.7% for IV) [12]. An imbalance in withdrawal of study treatment between groups at 20 months’ follow-up was attributed to cardiac disorders (3.0% [9/297] with SC trastuzumab versus 1.7% [5/298] with IV) but the rate of grade ≥3 cardiac AEs was low (5 [2%] vs 3 (1.0%)]. In the final analysis of HannaH (6 years’ follow-up), the incidence of cardiac AEs was similar in the SC and IV groups (14.8% vs 14.1%) and no new safety signals were identified [13].

**Fig. 4:**
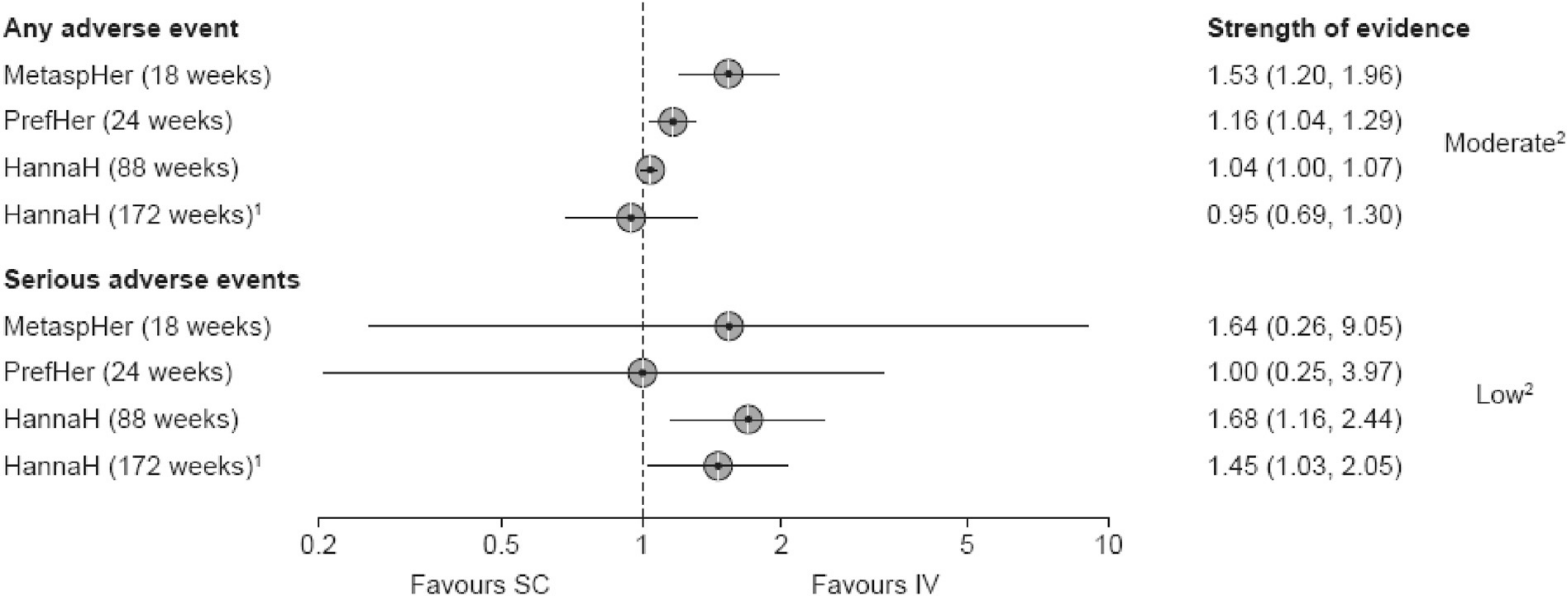
Forest plot of risk ratios for AEs and with SC vs IV trastuzumab (HannaH, PrefHer, and MetaPHER studies). Values are risk ratio and 95% confidence intervals. ^1^Drop-out rate 37%; ^2^Due to high risk of bias.

The rapid review of the HannaH, PrefHer [54,55] and MetaPher [56] studies reported a higher relative risk of experiencing an AE or SAE with SC versus IV trastuzumab (Fig. 4), due mainly to injection site-related AEs (e.g., pain and erythema) [57].

### 3.2 Serious adverse events

The rate of SAEs in the HannaH study was double with SC compared with IV trastuzumab (21% vs 12%), driven by a higher number of infections and infestations that required new or prolonged hospitalization (summarized in Supplementary Table 11) [10]. These events were manageable (resolving within a mean of 13 and 17 days in the IV and SC arms, respectively); however, the EMA pointed that the need for hospitalization or IV antibiotics for systemic infections is relevant from the patient’s perspective [4] and may compromise health-related quality of life (HRQL). These events also increase healthcare resource utilization, increasing costs. Importantly, the higher rate of infection-related SAEs and severe events with SC trastuzumab occurred during adjuvant treatment (i.e., during monotherapy), when the balance between benefit and risk of toxicity needs to be carefully considered, given that patients are not burdened by chemotherapy during this treatment phase [4].

### 3.3 Administration-related reactions

In HannaH, any-grade administration-related reaction (ARRs) occurred more frequently with SC than IV trastuzumab (48%, vs 37%) although the incidence of grade ≥3 reactions was low (around 2%) and there were no grade 4 ARRs [12]. The imbalance between arms was mostly due to disorders of the skin and SC tissue disorders or respiratory, thoracic, and mediastinal disorders [12].

Whilst late-onset ARRs might be expected with SC trastuzumab based on the PK profile [4], a retrospective single-center study did not find this to be the case [58]. Retrospective studies of SC trastuzumab have shown that ARRs with SC trastuzumab usually occur within 2 h of administration and are typically mild and self-limiting [[59], [60], [61]].

### 3.4 Injection-site reactions

Injection site reactions (ISRs) were experienced by 11% of patients receiving SC trastuzumab in the HannaH study [10]. While ISRs were mostly grade 1, avoiding these events may be relevant to patients who are already receiving other IV chemotherapies via a central line Indeed, in a real-world study in Germany, patients receiving chemotherapy or another IV antibody therapy in addition usually received IV trastuzumab, particularly if a central venous port was already in place, even though SC administration would have significantly reduced the time spent in the center by these patients. In these cases, avoiding additional injections was given preference over potential time savings [62].

### 3.5 Anti-drug antibodies

The incidence of trastuzumab ADAs was higher with SC than IV trastuzumab in the HannaH study (15% vs 7%), although neutralizing antibodies were detected in only two and one patients, respectively. The rate of rHuPH20 ADAs in the SC group was 16% (48/295). ADAs did not appear to affect PK, efficacy (pCR and EFS) or rate of ARRs [12].

### 3.6 Summary

Overall, SC administration of trastuzumab was associated with a higher rate of SAEs than IV administration [10], largely infections and infestations that required new or prolonged hospitalization [10], which may compromise HRQL and also incur healthcare costs. SAEs occurred largely during adjuvant treatment when patients are no longer burdened by chemotherapy. Thus, decisions on the appropriate route of administration should take into consideration the increased risk of SAEs, ISRs and ADAs with the SC formulation alongside potential benefits and patient preferences, particularly during adjuvant monotherapy [4] and in patients who already have a central line.

## 4 Study limitations

A scoping review is a validated method that allows mapping of disparate literature without the potential bias towards randomized controlled trials that is inherent in systematic review. This method is useful to identify issues in the literature that might be subsequently evaluated in a systematic review. Here we comment on the limits of the database in addressing the research questions.

The evidence base supporting the non-inferiority of SC trastuzumab is limited, originating solely from the prospective randomized HannaH study; no other clinical trials were identified in our systematic search.

All statistical comparisons for differences in AE rates between SC and IV trastuzumab in the HannaH study were descriptive.

In terms of immunogenicity, the HannaH study provided only a descriptive analysis whereas a recent narrative review reported a significant two-fold association between development of ADAs and receiving SC trastuzumab (p = 0.003; Fisher’s exact test) [63]. An association with neutralizing ADAs was not reported, and so far there has been no evidence that patients develop long-lasting memory ADAs or that this could affect the safety or efficacy of subsequent treatment with IV trastuzumab or pertuzumab [63]. ADA formation was monitored in the HannaH trial extension as an additional pharmacovigilance activity linked to the potential risk of immunogenicity [4]. The Phase 3b single-arm MetaPHER study, which evaluated the safety and efficacy of SC trastuzumab in combination with IV pertuzumab and docetaxel in the first-line treatment of HER2-positive MBC, reported a higher incidence of post-treatment ADAs to SC trastuzumab (24%) than in HannaH (15%), although this was not associated with an increased frequency or severity of ARRs [10,64]. In addition, a small observational study of 51 patients with non-metastatic HER2-positive breast cancer, published after this scoping review was completed, did not identify ADAs with IV (n = 24; median of 13 treatment cycles) or SC trastuzumab (n = 27; 10 cycles) [65]. However, these results should be interpreted with caution given the small sample.

## 5 Conclusions

This scoping review highlights the lack of data comparing IV and SC trastuzumab beyond the HannaH study. Real-world evidence suggests that a significant proportion of overweight/obese patients may not reach the target plasma concentration during the first cycle of treatment with 600 mg SC trastuzumab [37,38]. Given the prevalence of obesity in patients with breast cancer [19], this may be a concern in clinical practice, as dosing aims to reach the target concentration from the first cycle of treatment [10].

The scoping review did not identify evidence indicating that low-weight patients who receive a fixed SC dose of trastuzumab are at increased risk of cardiotoxicity.

In terms of safety, SC trastuzumab was associated with higher rates of infection-related and severe events requiring hospitalization, which affects patients and increases healthcare resource utilization and costs. Notably, these events occurred during adjuvant monotherapy when patients are not burdened by chemotherapy. The balance between benefit and risk of toxicity with SC versus IV trastuzumab therefore needs to be considered carefully in this setting [4]. The incidence of ADAs was also higher with SC than IV trastuzumab, although these were rarely neutralizing ADAs. Finally, SC trastuzumab was associated with ISRs in about 11% of patients in HannaH. When discussing treatment in a particular setting, the clinical benefit, risks and appropriate dosing of IV or SC trastuzumab should be discussed with the patient according to their individual needs.

## Funding

This work was funded by Amgen Inc.

## Declaration of competing interest

Hans-Christian Kolberg has received honoraria from Carl Zeiss meditec, Teva, Theraclion, Novartis, Amgen, AstraZeneca, Pfizer, Janssen-Cilag, GSK, LIV Pharma, Roche, MSD, SurgVision, Onkowissen, Daiichi Sankyo, and Genomic Health.

Christian Jackisch has received honorira from Amgen, AstraZeneca, Roche, Celgene, Pfizer, Novartis, Lilly, and Exact Sciences.

Sara Hurvitz has conducted research (fees paid to institution) for: Ambrx, Amgen, Arvinas, Bayer, Daiichi-Sankyo, Dignitana, Genentech/Roche, GSK, Immunomedics, Lilly, Macrogenics, Novartis, Pfizer, OBI Pharma, Pieris, PUMA, Radius, Sanofi and Seattle Genetics, and performs consultancy and holds stock options for NK Max.

Vladimir Hanes and Delphine Courmier are employees of Amgen, Inc.

Julie Winstone and Helen Barham have no declarations of interest.
