# Supplementary material for: Is weight-based IV dosing of trastuzumab preferable to SC fixed-dose in some patients? A systematic scoping review
Source: Breast. 2021 Mar 18;57:95–103. doi: 10.1016/j.breast.2021.03.003 (PMC8044716; doi:10.1016/j.breast.2021.03.003)
Supplement: Multimedia component 1 [file mmc1.docx]

# Methodology

The objectives of the scoping review were addressed using the PICOS (population, intervention, comparators, outcomes, study design/setting) criteria. The inclusion and exclusion criteria for the three review questions differ so the eligibility criteria are presented separately for each question in Table 1 (question 1), Table 2 (question 2), and Table 3 (question 3). Key differences between studies are in bold.

Table 1 Question 1 Eligibility Criteria

| Criteria | Include | Exclude |
| --- | --- | --- |
| **Population** | Interventional/observational studies:   - Adults (aged ≥18 years) receiving trastuzumab **within approved indications (e.g., HER2-positive early BC, metastatic BC, metastatic GC)**   PopPK analyses:   - Adults with cancer or healthy volunteers | Any patient population other than those listed |
| **Intervention** | Interventional/observational studies:   - **SC trastuzumab**   PopPK studies:   - **SC or IV trastuzumab** | Any treatment other than those listed (e.g., trastuzumab antibody-drug conjugates) |
| **Comparators** | **Interventional/observational studies:**   - **IV trastuzumab** | Any comparator other than IV trastuzumab (e.g., trastuzumab antibody-drug conjugates) |
| **Outcomes** | Interventional/observational studies:   - **Efficacy by body weight or trastuzumab exposure**   PopPK studies:   - **Influence of body weight on PK and/or exposure with trastuzumab** | Any outcomes other than those listed |
| **Study design/setting** | **Interventional studies (randomized and non-randomized controlled studies), observational studies (prospective, retrospective), popPK modeling studies** | - Editorials - Case reports - Studies with <10 patients - In vitro studies |
| **Language of publication** | English | Non-English |
| **Date of publication** | - Full publications: no restriction - Conference abstracts published from 2017 to 2020 inclusive | Conference abstracts prior to 2017 |
| **Countries** | No restriction | NA |

BC, breast cancer; GC, gastric cancer; IV, intravenous; popPK, population pharmacokinetics; SC, subcutaneous

Table 2 Question 2 Eligibility Criteria

| Criteria | Include | Exclude |
| --- | --- | --- |
| **Population** | Interventional/observational studies:   - Adults (≥18 years) with cancer **or healthy volunteers; the eligible patient population is broad because dose-escalation studies are often conducted in healthy adults or in patients with a variety of tumor types**   PopPK analyses:   - Adults with cancer **or healthy volunteers** | Any patient population other than those listed |
| **Intervention** | Interventional/observational studies:   - **IV trastuzumab**   PopPK studies:   - SC or IV trastuzumab | Any treatment other than those listed (e.g., trastuzumab antibody-drug conjugates) |
| **Comparators** | **Escalating doses of IV trastuzumab or multiple loading/maintenance doses of IV trastuzumab** | Any comparator other than those listed (e.g., trastuzumab antibody-drug conjugates) |
| **Outcomes** | Clinical trials:   - **Rate of cardiotoxicity for each dose of IV trastuzumab**   PopPK studies:   - **Simulated cardiotoxicity by exposure or body weight** | Any outcomes other than those listed |
| **Study design/setting** | **Early phase (1–2) trials evaluating escalating doses of IV trastuzumab; clinical studies comparing multiple loading/ maintenance doses of IV trastuzumab;** **popPK modeling studies** | - Studies evaluating single loading and maintenance doses of trastuzumab - Studies reporting risk factors for trastuzumab-associated cardiotoxicity (overweight patients typically have many comorbidities placing them at higher risk of cardiac AEs, which confounds any effect of low bodyweight/overdosing on cardiotoxicity) - Editorials - Case reports - Studies with <10 patients - In vitro studies |
| **Language of publication** | English | - Non-English |
| **Date of publication** | - Full publications: no restriction - Conference abstracts published from 2017 to 2020 inclusive | - Conference abstracts published before 2017 |
| **Countries** | No restriction | - NA |

AE, adverse event; IV, intravenous; popPK, population pharmacokinetics; SC, subcutaneous

Table 3 Question 3 Eligibility Criteria

| Criteria | Include | Exclude |
| --- | --- | --- |
| **Population** | **Adults receiving trastuzumab within approved indications for the SC formulation (e.g., HER2-positive early BC, metastatic BC)** | Any patient population other than those listed |
| **Intervention** | **Fixed dose SC trastuzumab 600 mg** | Any treatment other than those listed |
| **Comparators** | **IV trastuzumab** | Any comparator other than those listed (e.g., trastuzumab antibody-drug conjugates) |
| **Outcomes** | - **Adverse events** - **Administration-related reactions** - **Injection site reactions** - **Immunogenicity** | Any outcomes other than those listed |
| **Study design/setting** | **Interventional studies (randomized and non-randomized controlled trials), observational studies (prospective, retrospective)** | - **Single-arm studies of SC** trastuzumab - Editorials - Case reports - Studies <10 patients - In vitro studies |
| **Language of publication** | English | Non-English |
| **Date of publication** | - Full publications: no restriction - Conference abstracts published from 2017 to 2020 inclusive | Conference abstracts published prior to 2017 |
| **Countries** | No restriction | NA |

BC, breast cancer; GC, gastric cancer; IV, intravenous; popPK, population pharmacokinetics; SC, subcutaneous

Studies were identified by searching electronic databases, reference lists of relevant articles, conference proceedings (identified using the Conference Proceedings Citation Index [CPCI]). The search strategy for a scoping review is iterative, allowing additional search terms and sources identified throughout the review process to be incorporated into the search strategy. As recommended in the JBI framework for conducting scoping reviews, a three-step search strategy was implemented in this scoping review.

- Step 1: An Initial search of two appropriate online databases relevant to the questions of interest should be conducted to identify keywords and index terms used to describe the articles. The electronic databases MEDLINE (via PubMed) and Web of Science were searched using broad terms and phrases (e.g., subcutaneous AND trastuzumab, trastuzumab AND “population pharmacokinetic”, trastuzumab AND (phase I OR phase 2 or dose-finding/escalation/expansion/ranging)). Relevant review articles and selected publications of primary studies were then searched to identify controlled vocabulary (e.g., MeSH terms for PubMed), keywords, free text words, and index terms). These terms were then entered in PubMed to identify additional related terms and word variations (synonyms).
- Step 2: All MeSH terms, keywords, text words, and index terms identified from Step 1 were used to develop the core search strategy for PubMed (Table 4). This search strategy was designed to identify the primary literature base for the three review questions. The search strings were then modified to account for differences in syntax and thesaurus headings for searches of the Cochrane Library (Table 5), SCOPUS (Table 6) and Web of Science (Table 7).
- Step 3: The grey literature was searched to identify further studies not captured in the database searches. The CPCI was searched to identify relevant abstracts and posters from proceedings of conferences held between 2017 and 2020 inclusive. Reference lists of included publications and relevant SLRs/NMAs and narrative reviews were hand searched. The websites of the FDA and EMA were searched to identify relevant regulatory documents for SC trastuzumab.

The following data sources were searched:

- Electronic databases:
- MEDLINE® (PubMed), 1946 to present day
- The Cochrane library, incorporating the Cochrane Central Register of Controlled Trials (CENTRAL) and the Cochrane Database of Systematic Reviews (Cochrane Reviews)
- SCOPUS (largest abstract and citation database of peer-reviewed literature from 2004 to present day)
- Conference proceedings: CPCI via Web of Science (Clarivate Analytics)
- Websites of the FDA (<https://www.accessdata.fda.gov/scripts/cder/daf/>) and EMA (<https://www.ema.europa.eu/en>).
- Reference lists of included publications and relevant SLRs/MAs.

The project lead (JMW) conducted the searches. Results from the database searches were downloaded into an EndNote library, which was used to manage citation screening; duplicate citations were removed. The titles, and abstracts when available, were reviewed by two independent researchers (JMW and HB; first pass). Full publications of studies deemed potentially relevant at this stage were obtained and examined by two researchers (JMW and HB; second pass). Final inclusion and exclusion of citations was verified by the project lead (JMW). Disputes regarding eligibility were resolved through discussion.

The reasons for exclusion following review of full publications (second pass) were documented, both descriptively and using a prospectively designed code system. The reasons for exclusion at second pass were also summarized in a PRISMA flow diagram.[^1^](#_ENREF_1)

On completion of the scoping review, a list of potentially relevant citations was shared with Amgen for review. The final list of studies for inclusion in the review was then agreed.

A data extraction sheet was created in Microsoft® Excel to chart the data from studies that met the eligibility criteria. Data extraction was conducted by the project lead (JMW), and quality checked for 100% of data elements by a senior reviewer (HB). Disputes were resolved through discussion. Relevant clinical trial publications and conference abstracts were also extracted into the data extraction table. Systematic literature reviews and meta-analyses were summarized in the report.

The findings of the scoping review were summarized in a report as per PRISMA reporting guidelines and a narrative synthesis and summary tables of the evidence developed.

Table 4 Search of MEDLINE (PubMed): Date of search, 18 January 2020

| # | Searches | Results |
| --- | --- | --- |
| 1 | trastuzumab[MeSH Terms] OR trastuzumab[All Fields] OR herceptin[All Fields] OR trastuzumab's[All Fields] | 11,390 |
| 2 | subcutan*[All Fields] OR fixed dos*[All Fields] OR injections, subcutaneous[MeSH Terms] | 197,996 |
| 3 | #1 AND #2 | 214 |
| 4 | clinical trial, phase i[Publication Type] OR clinical trial, phase ii[Publication Type] OR clinical trials, phase i as topic[MeSH Terms:noexp] OR clinical trials, phase ii as topic[MeSH Terms:noexp] OR (dose[TIAB] AND (ranging[TIAB] OR finding[TIAB] OR escalation[TIAB] OR expansion[All Fields] OR intensive[TIAB])) | 115,100 |
| 5 | #1 AND #4 | 647 |
| 6 | (population[TIAB] AND pharmacokinetic*[TIAB]) OR (pharmacokinetic*[TIAB] AND model*[TIAB]) OR (nonlinear[TIAB] AND mixed[TIAB] AND effect*[TIAB]) OR (non-linear[TIAB] AND mixed[TIAB] AND effect*[TIAB]) OR NONMEN[TIAB] OR ((covariate[TIAB] OR covariates[TIAB] OR covariation[TIAB] OR covariational[TIAB] OR covariations[TIAB]) AND analys*[TIAB]) | 82,383 |
| 7 | #1 AND #6 | 161 |
| 8 | #3 OR #5 OR #7 | 972 |
| 9 | Case reports[publication type] OR editorial[publication type] | 2,581,482 |
| 10 | #8 NOT #9 | 953 |
| 11 | #8 NOT #9 Filters: English | 929 |

Table 5 The Cochrane Library including Cochrane Central Register of Controlled Trials (CENTRAL), Cochrane Database of Systematic Reviews (Cochrane Reviews): Date of search, 20 January 2020

| # | Searches | Results |
| --- | --- | --- |
| 1 | MeSH descriptor: [Trastuzumab] this term only | 563 |
| 2 | trastuzumab OR Herceptin | 2614 |
| 3 | subcutaneous* | 30191 |
| 4 | (#1 OR #2) AND #3 | 101 |
| 5 | MeSH descriptor: [Clinical Trials, Phase II as Topic] explode all trees | 132 |
| 6 | MeSH descriptor: [Clinical Trials, Phase I as Topic] explode all trees | 60 |
| 7 | (phase AND (i OR ii OR 1 OR 2)):ti,ab,kw OR (dose AND (finding OR ranging OR escalation OR expansion OR intensive)):ti,ab,kw | 154102 |
| 8 | #5 OR #6 OR #7 | 154102 |
| 9 | (#1 OR #2) AND #8 | 1471 |
| 10 | ("population pharmacokinetic" OR "population pharmacokinetics" OR "nonlinear mixed effect" OR "nonlinear mixed effects" OR "non-linear mixed effect" OR "non-linear mixed effects" OR "covariate analysis" OR "covariate analyses" OR nonmen ) | 2297 |
| 11 | (#1 OR #2) AND #10 | 17 |
| 12 | #4 OR #9 OR #11 | 1530 |

Table 6 Search of SCOPUS: Date of search, 20 January 2020

| **#** | **Searches** | **Results** |
| --- | --- | --- |
| 1 | TITLE-ABS ( ( trastuzumab OR herceptin ) AND subcutan* ) | 204 |
| 2 | TITLE-ABS ( ( trastuzumab OR herceptin ) AND ( "phase i" OR "phase ii" OR "phase 1" OR "phase 2" OR ( dose AND ( finding OR ranging OR escalation OR expansion OR intensive ) ) ) ) | 993 |
| 3 | TITLE-ABS ( ( trastuzumab OR herceptin ) AND ( "population pharmacokinetic" OR "population pharmacokinetics" OR "nonlinear mixed effect" OR "nonlinear mixed effects" OR "non-linear mixed effect" OR "non-linear mixed effects" OR "covariate analysis" OR "covariate analyses" OR nonmen ) ) | 27 |
| 4 | #1 OR #2 OR #3 | 1,187 |
| 5 | #3 AND NOT INDEX (medline) | 246 |
| 6 | #5 AND ( EXCLUDE ( DOCTYPE , "ch" ) ) | 242 |
| 7 | #6 AND ( LIMIT-TO ( LANGUAGE , "English" ) ) | 208 |

Table 7 Search of Web of Science: Date of search, 20 January 2020

| **#** | **Searches** | **Results** |
| --- | --- | --- |
| 1 | TOPIC: (Subcutan* AND (trastuzumab OR Herceptin)  Databases= WOS, BCI, BIOSIS, CCC, DRCI, DIIDW, KJD, MEDLINE, RSCI, SCIELO, ZOOREC Timespan=All years  Search language=Auto | 434 |
| 2 | TOPIC: (TOPIC: (trastuzumab OR herceptin) AND TOPIC: ("population pharmacokinetic" OR "population pharmacokinetics" OR "nonlinear mixed effect" OR "nonlinear mixed effects" OR "non-linear mixed effect" OR "non-linear mixed effects" OR "covariate analysis" OR "covariate analyses" OR NONMEN)  Databases= WOS, BCI, BIOSIS, CCC, DRCI, DIIDW, KJD, MEDLINE, RSCI, SCIELO, ZOOREC Timespan=All years  Search language=Auto | 11 |
| 3 | TOPIC: (trastuzumab OR Herceptin) AND ("phase i" OR "phase 1" OR "phase ii" OR "phase 2") OR (dose AND (finding OR ranging OR escalation OR expansion OR intensive))  Databases= WOS, BCI, BIOSIS, CCC, DRCI, DIIDW, KJD, MEDLINE, RSCI, SCIELO, ZOOREC Timespan=All years  Search language=Auto | 4,625 |
| 4 | #3 OR #2 OR #1  Databases= WOS, BCI, BIOSIS, CCC, DRCI, DIIDW, KJD, MEDLINE, RSCI, SCIELO, ZOOREC Timespan=All years  Search language=Auto | 4,984 |
| 5 | #3 OR #2 OR #1  Refined by: [excluding] Databases: ( MEDLINE )  Databases= WOS, BCI, BIOSIS, CCC, DRCI, DIIDW, KJD, MEDLINE, RSCI, SCIELO, ZOOREC Timespan=All years  Search language=Auto | 1,295 |
| 6 | #3 OR #2 OR #1  Refined by: [excluding] Databases: ( MEDLINE ) AND LANGUAGES: ( ENGLISH )  Databases= WOS, BCI, BIOSIS, CCC, DRCI, DIIDW, KJD, MEDLINE, RSCI, SCIELO, ZOOREC Timespan=All years  Search language=Auto | 1,247 |
| 7 | #3 OR #2 OR #1  Refined by: [excluding] Databases: ( MEDLINE ) AND LANGUAGES: ( ENGLISH ) AND [excluding] DOCUMENT TYPES: ( BOOK OR PATENT OR EDITORIAL )  Databases= WOS, BCI, BIOSIS, CCC, DRCI, DIIDW, KJD, MEDLINE, RSCI, SCIELO, ZOOREC Timespan=All years  Search language=Auto | 1,159 |

# Question 1 summary tables

Table 8 Population pharmacokinetic models for trastuzumab included in the scoping review

| Article | Data source | Route | Population, n (PK samples) | Baseline BW, median (range) | PopPK model | Influence of BW on point estimate | Influence of BW on PK |
| --- | --- | --- | --- | --- | --- | --- | --- |
| Bernadou et al. (2016)[^2^](#_ENREF_2) | Phase 2 RADHER study (NCT00674414) | IV | HER2+ EBC  n = 79 (784) | 61  (48–147) | 2C model (MONOLIX), first-order transfer and elimination rate constants | - Vc: 0.7 (RSE 23%) - CL: 0.56 (19%) | - V1 increased with BW (LRT = 18.9, p = 1.4 × 10^-5^): - CL increased with BW (LRT = 27.6, p = 1.5 × 10^-7^) |
| Bruno et al. (2005)[^3^](#_ENREF_3) | Phase 1, 2, and 3 studies | IV | HER2+ MBC; advanced solid tumors  n = 476 (3,249) | 70  (42–119) | 2C model (NONMEM) | - Vc: 0.556 (95% CI 0.211–0.824) | - BW second most influential covariate for Vc - Vc 2.5–3.7 L in patients with extreme BW (i.e., 49–96 kg [5^th^–95th percentile]) |
| Charoin et al. (2004)[^4^](#_ENREF_4) | Phase 2, 3 studies | IV | HER2+ MBC  n = 194 (2,508) | n/r | 2C model (NONMEM) | - CL: 0.562 (CV 25%) | - BW only significant covariate for CL |
| Chen et al. (2019)[^5^](#_ENREF_5) | Phase 3 study of biosimilar trastuzumab (NCT01989676) | IV | HER2+ MBC  n = 707 (n/r) | 67.0  (29–147)^a^ | 2C model (NONMEM) with first-order elimination, zero-order input | Biosimilar:   - Vc: 0.507 (95% CI 0.316–0.698) - CL: 0.637 (0.450–0.824)   Originator:   - Vc: 0.512 (0.026–0.998) - CL: 0.673 (0.430–0.916) | - BW significant covariate for V1 and CL for both biosimilar and originator trastuzumab |
| Cosson et al. (2014)[^6^](#_ENREF_6) | Phase 3 ToGA study  (NCT01041404) | IV | HER2+ GC (96% MGC)  n = 266 (1,419) | 62.0 (35.3–111.0) | 2C model (NONMEM), parallel first-order linear and non-linear elimination | - Vc: 0.648 (RSE 12.4%) - CL: 1.07 (RSE 16.9%) | - Patients with the lowest weight (35.3 kg) had 45.3% lower CL than typical CL for a 62 kg patient - Patients with highest body weight (111 kg) had 86.5% increase in CL vs typical CL - Vc reduced by 30.6% (vs typical Vc) in lowest weight patients and increased by 45.8% in highest weight patients |
| Fukishima et al. (2007)[^7^](#_ENREF_7) | 5 Phase 1–3 | IV | HER2+ EBC, MBC  n = 265 (3,979) | n/r | 2C model (NONMEM), log normal inter-patient variability | - Vc: 0.484 (95% CI 0.329–0.639) - CL: 0.557 (0.312–0.802) | n/r (poster only) |
| Hourcade-Potelleret et al. (2015)[^8^](#_ENREF_8) | Phase 1/1b trial; validated using Phase 3 HannaH | SC, IV | HMVs, HER2+ EBC  n = 66 (786) | n/r | 2C model (NONMEM), linear and non-linear elimination  Model used to simulate PK profile for four fixed SC doses (400, 500, 600, 700 mg Q3W) | - CL: 0.591 (Fix) - Vc: 0.48 (Fix) | - 600 mg was lowest dose for which 5^th^ percentile, median, and 95^th^ percentile for Cycle 1 and 7, Ctrough, and Cycle 7 AUC were ≥ values with IV dosing |
| Quartino et al. (2016)[^9^](#_ENREF_9) | Phase 3 HannaH study | SC (syringe) vs IV | HER2+ EBC  n = 592  (15,761) | SC: 68 (39-136)^b^  IV: 66 (42–137.1)^b^ | 2C model (NONMEM), linear and non-linear elimination | - Vc: 0.443 (RSE 11.3%) - Vp: 0.500 (22.2%) - CL: 1.04 (11.3%) | - Increased BW resulted in increased CL and Vc - BW explained 8% of variability in linear CL, 10% in Vc, and 28% in Vp |
| Quartino et al. (2019)[^10^](#_ENREF_10) | Phase 1–3 trials | IV | HER2+ EBC, MBC, AGC, NSCLC, other tumors, HMVs  n = 1,582 (26,040) | 66 | 2C model (NONMEM), linear and non-linear elimination | CL: 0.967 (RSE 7.19%) | - BW most important covariate for CL - Compared with CL for a 66 kg patient, linear CL decreased 27% and increased 43% for 46 kg and 98 kg, respectively |
| Reijers et al. (2016)[^11^](#_ENREF_11) | Phase 1 study of trastuzumab biosimilar (FTMB; Synthon BV) | IV | HMVs  110 (1,247) | Mean: 77.2c  (SD: 10.6) | 3C model (NONMEM), central [V1], peripheral [V2, V3], linear and non-linear elimination | n/r | Combined (biosimilar and originator) model:   - Significant correlations between LBW (0.61), BW (0.55), BSA (0.60), height (0.54), and BMI (0.28) with V1 - Linear regression analysis:   LBW vs BSA: 1  LBW vs BW: 0.96   - BMI was most significant covariate for linear CL (k_e_, 0.6) and improved model - LBW as linear covariate for V1 significantly improved model OFV (not shown; adding other weight-related covariates did not improve model vs LBW) |

^a^Median body weight reported in publication for Phase 3 study (Pegram et al. 2019[^12^](#_ENREF_12)as baseline characteristics not reported in popPK study.

bMedian body weight reported in publication for Phase 3 HannaH study (Jackisch et al. 2016[^13^](#_ENREF_13) as baseline characteristics not reported in popPK study.

^c^Mean body weight reported in publication for Phase 1 study (Wisman et al. 2014[^14^](#_ENREF_14) as baseline characteristics not reported in popPK study.

AUC, area under plasma concentration–time curve; BSA, body surface area; 2C, two-compartment; 3C, three-compartment; AGC, advanced gastric cancer; BW, body weight; CI, confidence interval; CL, clearance; C_trough_, trough plasma concentration; EBC, early breast cancer; GC, gastric cancer; HMV, healthy male volunteers; HER2, human epidermal growth factor receptor 2; IV, intravenous; LBW, lean body weight;; LRT, likelihood ratio test; MBC, metastatic breast cancer; MGC, metastatic gastric cancer; NONMEM, non-linear mixed effects modeling; n/r, not reported; NSCLC, non-small cell lung cancer; OFV, objective function value; PK, pharmacokinetics; RSE, relative standard error; SC, subcutaneous; Vc/V1, volume distribution of the central compartment; V2/V3, peripheral compartment volume

Table 9 Dose-escalation studies of IV trastuzumab included in the scoping review

| Reference | Study design and setting | Population (n) | Treatment and doses | Key finding(s) |
| --- | --- | --- | --- | --- |
| Fleming (2002)[^15^](#_ENREF_15) | - Phase 1 dose-escalation study of IV trastuzumab + SC IL-2 - Multicenter, US | HER2+ solid tumors  (n = 45) | - 1, 2, 4 and 8 mg/kg weekly or 8 mg/kg biweekly, escalated in cohorts of >6 patients - Low-dose SC IL-2 daily | - No cardiotoxicity - No dose-related toxicity |
| Safran (2004)[^16^](#_ENREF_16) | - Phase 1 non-randomized, dose-escalation study of IV trastuzumab + chemoradiotherapy - Multicenter, US | HER2+ and HER2–esophageal cancer  (n = 30) | - Bolus of 2, 3 or 4 mg/kg then 1, 1.5 or 2 mg/kg weekly, respectively, for 5 weeks (prior to chemotherapy) - Paclitaxel, cisplatin, radiotherapy | - No Grade ≥ 3 cardiotoxicity - Mean LVEF was 57% before treatment and 56% after treatment |
| Tokuda (1999)[^17^](#_ENREF_17) | - Phase 1 dose-escalation study of IV trastuzumab monotherapy - Japan | HER2+ MBC  (n = 18) | - 3 patients at each dose level - Starting dose 1 mg/kg, escalated to 2, 4 8 mg/kg - First dose followed after 3 weeks with 9 weekly doses | - No grade ≥3 cardiotoxicity at any dose |
| Wynne (2013)[^18^](#_ENREF_18) | - Phase 1b non-randomized, OL dose-finding/ confirmation study (NCT00800) of SC trastuzumab - 2 centers in New Zealand and Australia | HMVs; HER2+ EBC  (Part 1: n = 30; Part 2: 40) | Part 1 (dose-finding), single dose:   - Cohort (C)1: 6 mg/kg IV (HMVs) - C2: 6 mg/kg IV (patients) - C3: 6 mg/kg SC (HMVs) - C4: 10 mg/kg SC (HMVs) - C5: 8 mg/kg (HMVs)   Part 2 (dose-confirmation), single dose (patients)   - CA: 8 mg/kg SC - CB: 12 mg/kg SC | - No cardiotoxicity - No increase in incidence or severity of AEs with escalating SC dose of trastuzumab among HMVs or patients |
| Zhou(2015)[^19^](#_ENREF_19) | - Phase 1, OL, randomized dose-escalation study of biosimilar IV trastuzumab (Cipterbin) - Single center, China | MBC  (n = 27) | - Escalating single doses of 100, 250, and 500 mg biosimilar trastuzumab (9 patients per dose level) | - 1 patient had Grade 4 reversible sinus bradycardia after single 250 mg dose (6.25 mg/kg) - Other cardiotoxicities were mild and not trastuzumab-related - No significant decrease in LVEF; no CHF |

AE, adverse event; CHF, congestive heart failure; EBC, early breast cancer; HMVs, health male volunteers; IL-2, interleukin-2; IV, intravenous; LVEF, left ventricular ejection fraction; MBC, metastatic breast cancer; OL, open label; SC, subcutaneous

Table 10 Studies comparing different loading doses of IV trastuzumab included in the scoping review

| Reference | Study design and setting | Population (n) | Treatment and doses | Key finding(s) |
| --- | --- | --- | --- | --- |
| Leyland-Jones (2010)[^20^](#_ENREF_20) | - Phase 1–2 single-arm OL study of IV trastuzumab with intensive loading dose - Multicenter (Spain, Canada, UK) | HER2+ MBC  (n = 72) | - 6 mg/kg loading dose on days 1, 8, and 15 of cycle 1 then 6 mg/kg maintenance dose Q3W | - LVEF decrease reported as an AE in 2 patients - LVEF events (n)   Increase/no change: 12  Decrease <15 pp: 44  Decrease ≥15 pp: 5  Decrease to <40%: 1 |
| Shah (2017)[^21^](#_ENREF_21) | - Phase 3b OL randomized study (HELLOISE) of SoC vs HD IV trastuzumab - International (23 countries), multicenter | HER2+ MGC/GEJ cancer  (n = 248) | - HD: 8 mg/kg loading dose then 10 mg/kg maintenance dose Q3W - SoC: 8 mg/kg loading dose then 6 mg/kg maintenance dose Q3W - Up to 6 cycles of chemotherapy (capecitabine and cisplatin) | - Serious cardiac disorders:   SoC: 3 (2.4%)  HD: 4 (3.3%) |
| Xu (2014)[^22^](#_ENREF_22) | - Phase 1b, single-arm OL study (NCT00927589) of off-label accelerated loading dose of IV trastuzumab - Multicenter, US | HER2+ locally advanced or metastatic solid tumors  (n = 59) | - 6 mg/kg on cycle 1 days 2 and 8 then 6 mg/kg Q3W | - Trastuzumab had no clinically relevant effect on QT interval - 8 patients had cardiac AEs   None discontinued treatment due to cardiac AEs   - Mean LVEF: 64% at baseline vs 59.5% post-baseline   No post-baseline LVEF values <45% |

AE, adverse event; GEJ, gastroesophageal junction; HD, high dose; IV, intravenous; LVEF, left ventricular ejection fraction; MBC, metastatic breast cancer; MGC, metastatic gastric cancer; OL, open label; pp, percentage points; Q3W, every three weeks; SoC, standard of care

Table 11 Rate of infections reported in clinical studies comparing SC and IV trastuzumab

|  |  | Any grade | | | Grade ≥3 | | | SAEs | | |
| --- | --- | --- | --- | --- | --- | --- | --- | --- | --- | --- |
| Trial, population, n | Follow-up | SC | IV | SC | | IV | SC | | IV |  |
| HannaH[^23^](#_ENREF_23)^,^[^24^](#_ENREF_24)   - HER2+ EBC (NA-A) (n = 596) | 1-year treatment period | n/r | n/r | 24 (8.1) | | 13 (4.4) | 24 (8.1) | | 13 (4.4) |  |
|  | 20 months | n/r | n/r | 21 (7.1)  NA: 10 (3.4)  A: 11 (3.7) | | 15 (5.0)  NA: 9 (3.0)  A: 6 (2.0) | 24 (8.1)  NA: 10 (3.4)  A: 13 (4.4) | | 13 (4.4)  NA: 8 (2.7)  A: 5 (1.7) |  |
| PrefHer[^25^](#_ENREF_25)   - HER2+ EBC (trastuzumab monotherapy after CT) (n = 488) | Cycle 1–4 (before crossover); (SC: 243; IV: 240) | 25 (10.3) | 23 (9.6) | n/r | | n/r | n/r | | n/r |  |
| MetaspHer[^26^](#_ENREF_26)   - HER2+ MBC (after long-term response with first-line trastuzumab) (n = 114) | Crossover period; pooled data;  (SC: 108; IV: 111) | 20 (18.5) | 14 (12.6) | 2 (1.9) | | 1 (0.9) | n/r | | n/r |  |

Values are number of patients (%)

A, adjuvant; CT, chemotherapy; EBC, early breast cancer; IV, intravenous; MBC, metastatic breast cancer; NA, neoadjuvant; n/r, not reported; SC, subcutaneous; SAE, serious advert event

References

1. Moher D *et al.* (2009) Preferred reporting items for systematic reviews and meta-analyses: the PRISMA statement*.* PLoS medicine 6: e1000097-e97.

2. Bernadou G *et al.* (2016) Influence of tumour burden on trastuzumab pharmacokinetics in HER2 positive non-metastatic breast cancer*.* British journal of clinical pharmacology 81: 941-48.

3. Bruno R *et al.* (2005) Population pharmacokinetics of trastuzumab in patients with HER2+ metastatic breast cancer*.* Cancer Chemother Pharmacol 56: 361‐69.

4. Charoin J-E *et al.* (2004) Population pharmacokinetic analysis of trastuzumab (Herceptin) following long-term administration using different regimens*.* Population Approach Group in Europe (PAGE) 13: Abstract 489.

5. Chen X *et al.* (2019) Population pharmacokinetics of PF-05280014 (a trastuzumab biosimilar) and reference trastuzumab (Herceptin®) in patients with HER2-positive metastatic breast cancer*.* Cancer Chemother Pharmacol 84: 83-92.

6. Cosson VF *et al.* (2014) Population pharmacokinetics and exposure-response analyses of trastuzumab in patients with advanced gastric or gastroesophageal junction cancer*.* Cancer Chemother Pharmacol 73: 737-47.

7. Fukushima Y *et al.* 2007 Abstrr 1121 Population pharmacokinetic analysis of trastuzumab (Herceptin) based on three different dosing regimens. . In: *16th Annual Meeting of the Population Approach Group in Europe (PAGE)*. PAGE: Copenhagen, Denmark. p. 16.

8. Hourcade-Potelleret F *et al.* (2014) Use of a population pharmacokinetic approach for the clinical development of a fixed-dose subcutaneous formulation of trastuzumab*.* CPT Pharmacometrics Syst Pharmacol 3: e87-e87.

9. Quartino AL *et al.* (2016) Population pharmacokinetic and exposure-response analysis for trastuzumab administered using a subcutaneous "manual syringe" injection or intravenously in women with HER2-positive early breast cancer*.* Cancer Chemother Pharmacol 77: 77-88.

10. Quartino AL *et al.* (2019) Population pharmacokinetic and covariate analyses of intravenous trastuzumab (Herceptin®), a HER2-targeted monoclonal antibody, in patients with a variety of solid tumors*.* Cancer Chemother Pharmacol 83: 329-40.

11. Reijers JAA *et al.* (2016) Use of population approach non-linear mixed effects models in the evaluation of biosimilarity of monoclonal antibodies*.* European journal of clinical pharmacology 72: 1343-52.

12. Pegram MD *et al.* (2019) PF-05280014 (a trastuzumab biosimilar) plus paclitaxel compared with reference trastuzumab plus paclitaxel for HER2-positive metastatic breast cancer: a randomised, double-blind study*.* British journal of cancer 120: 172-82.

13. Jackisch C *et al.* (2016) HannaH phase III randomised study: Association of total pathological complete response with event-free survival in HER2-positive early breast cancer treated with neoadjuvant-adjuvant trastuzumab after 2 years of treatment-free follow-up*.* Eur J Cancer 62: 62-75.

14. Wisman LAB *et al.* (2014) A phase I dose-escalation and bioequivalence study of a trastuzumab biosimilar in healthy male volunteers*.* Clin Drug Investig 34: 887-94.

15. Fleming GF *et al.* (2002) A phase I trial of escalating doses of trastuzumab combined with daily subcutaneous interleukin 2: report of cancer and leukemia group B 9661*.* Clinical cancer research : an official journal of the American Association for Cancer Research 8: 3718-27.

16. Safran H *et al.* (2004) Trastuzumab, paclitaxel, cisplatin, and radiation for adenocarcinoma of the esophagus: a phase I study*.* Cancer investigation 22: 670-77.

17. Tokuda Y *et al.* (1999) Dose escalation and pharmacokinetic study of a humanized anti-HER2 monoclonal antibody in patients with HER2/neu-overexpressing metastatic breast cancer*.* British journal of cancer 81: 1419-25.

18. Wynne C *et al.* (2013) Comparison of subcutaneous and intravenous administration of trastuzumab: a phase I/Ib trial in healthy male volunteers and patients with HER2-positive breast cancer*.* J Clin Pharmacol 53: 192-201.

19. Zhou X *et al.* (2015) A phase I dose-escalation study of a biosimilar trastuzumab in Chinese metastasis breast cancer patients*.* SpringerPlus 4: 1-6.

20. Leyland-Jones B *et al.* (2010) Intensive loading dose of trastuzumab achieves higher-than-steady-state serum concentrations and is well tolerated*.* Journal of clinical oncology : official journal of the American Society of Clinical Oncology 28: 960-66.

21. Shah MA *et al.* (2017) HELOISE: phase IIIb Randomized Multicenter Study Comparing Standard-of-Care and Higher-Dose Trastuzumab Regimens Combined With Chemotherapy as First-Line Therapy in Patients With Human Epidermal Growth Factor Receptor 2-Positive Metastatic Gastric or Gastroesophageal Junction Adenocarcinoma*.* Journal of clinical oncology 35: 2558‐67.

22. Xu N *et al.* (2014) Trastuzumab, in combination with carboplatin and docetaxel, does not prolong the QT interval of patients with HER2-positive metastatic or locally advanced inoperable solid tumors: results from a phase Ib study*.* Cancer Chemother Pharmacol 74: 1251-60.

23. Ismael G *et al.* (2012) Subcutaneous versus intravenous administration of (neo)adjuvant trastuzumab in patients with HER2-positive, clinical stage I-III breast cancer (HannaH study): a phase 3, open-label, multicentre, randomised trial*.* Lancet Oncol 13: 869-78.

24. Jackisch C *et al.* (2015) Subcutaneous versus intravenous formulation of trastuzumab for HER2-positive early breast cancer: updated results from the phase III HannaH study*.* Ann Oncol 26: 320-25.

25. Gligorov J *et al.* (2017) Switching between intravenous and subcutaneous trastuzumab: Safety results from the PrefHer trial*.* Breast 34: 89-95.

26. Pivot X *et al.* (2017) Patients' preference of trastuzumab administration (subcutaneous versus intravenous) in HER2-positive metastatic breast cancer: Results of the randomised MetaspHer study*.* Eur J Cancer 82: 230-36.
